# Effects of COVID-19 outbreak on Korean adolescents: Impact of altered economic perception on physical activity, sedentary behavior, and stress levels in an age-, gender-, and BMI-matched study

**DOI:** 10.1371/journal.pone.0294270

**Published:** 2023-11-13

**Authors:** Jisu Kim, In-Whi Hwang, Jeong-Hui Park, Youngdeok Kim, Jung-Min Lee

**Affiliations:** 1 Department of Kinesiology and Health Science, Virginia Commonwealth University, Richmond, Virginia, United States of America; 2 Graduate School of Physical Education, Kyung Hee University (Global Campus), Giheung-gu, Yongin-si, Gyeonggi-do, Republic of Korea; 3 Sports Science Research Center, Kyung Hee University (Global Campus), Giheung-gu, Yongin-si, Gyeonggi-do, Republic of Korea; 4 School of Public Health, Texas A&M Health Science Center, Bryan, Texas, United States of America; Universidade de Tras-os-Montes e Alto Douro Escola de Ciencias da Vida e do Ambiente, PORTUGAL

## Abstract

The current study is to examine the disparities in physical activity (PA), sedentary behavior (SB), and stress levels in Korean adolescents concerning changes in their perception of family economic status (ES) during COVID-19. Among a total of 6144 Korean adolescents aged 12 to 18, the participants were categorized into two groups based on their responses regarding changes in their family ES due to COVID-19: Declined ES (n = 3072) and Non-changed ES (n = 3072), with matching in terms of age, gender, and BMI. All variables were assessed using the 16^th^ year (2020) of the Korean Youth Risk Behavior Survey. Statistical analyses were conducted using the SPSS 26.0 version, employing independent *t*-tests to examine anthropometrics’ differences and multinominal logistic regression to predict the impact of perception of family ES on PA, SB, and stress while comparing the two groups. The significance level was set at α = 0.05. Adolescents in the Declined ES group were 1.2 times more likely to engage in MVPA for less than 420 mins/wk (OR = 1.16, *p* = 0.039), 1.7 times more likely to meet recommended muscular strength activities (i.e., ≥ 3 days/wk) (OR = 1.70, *p* < 0.001), 37% less likely to not meet recommended recreational sitting time (i.e., ≥ 840 mins/wk) (OR = 0.63, *p* < 0.01), and were 2.1 times more likely to experience very severe stress level than the Non-changed ES group (*p* < 0.001). These results shed light on the importance of promoting mental health care in adolescents, regardless of PA levels, for their well-being during potential future pandemics. Understanding the impact of perceived ES changes on health behaviors can inform targeted interventions and support strategies to improve the mental health outcomes of adolescents during challenging times.

## 1. Introduction

Coronavirus disease 2019 (COVID-19) is an epidemic disease associated with severe respiratory syndromes, and in 2019, the World Health Organization (WHO) declared it a global public health emergency [1]. To curb the spread of the COVID-19 virus, social distancing measures were implemented worldwide during the pandemic, leading to the closure of schools and public facilities and limiting non-essential gatherings [2–4].

Despite their effectiveness in reducing virus transmission, these necessary measures have unintended health-related consequences for children and adolescents. Specifically, the restrictions have resulted in decreased participation in physical activity (PA) [5–8], increased sedentary behavior (SB) [8–10], and disrupted sleep patterns [8, 11, 12] among the youth. Such reductions in PA and increased SB carry significant risks for the long-term health of young individuals, potentially contributing to the development of various chronic diseases that may persist into adulthood [13]. In light of these concerns, the WHO issued guidelines in 2020, recommending that children and adolescents engage in a minimum of 60 minutes or more of moderate-to-vigorous PA (MVPA) daily, including regular aerobic and muscle-strengthening activities at least three days a week [14, 15]. Unfortunately, the COVID-19 outbreak and the subsequent restrictions have limited the opportunities for youth to engage in PA-related activities [16].

In addition to its direct impact on the youth’s health, the COVID-19 pandemic and the consequent restrictions have had a substantial effect on families’ economic status (ES), including the economic crisis, income loss, and unemployment [17]. These socioeconomic changes are indirectly associated with the health-related behaviors of the youth, and extensive research has consistently demonstrated significant associations between family ES and health-related behaviors among youth. Studies have revealed that adolescents from high-income families tend to engage in more MVPA and experience lower levels of inactivity compared to counterparts from low-income families [18–20]. Conversely, research has indicated that children from low-ES families face a higher risk of various health-related issues, such as sleep deficiency, overweight, academic stress, and other mental health problems [21–24]. Additionally, several studies have emphasized the significant association between parental support and youth engagement in PA [25–27]. Considering this existing evidence, it is reasonable to hypothesize that the socioeconomic impact of COVID-19 may influence children’s daily PA, possibly mediated by parental support [5].

In light of the importance of exploring the interplay between external factors, family processes, and children’s perceptions [28, 29], it becomes essential to consider the impact of COVID-19 on family ES and how it may have influenced children’s perception of their family’s ES, irrespective of the actual family ES. Thus, alongside actual family ES, gaining insight into children’s perception of their family ES becomes imperative to fully comprehend the multifaceted relationship between socioeconomic factors and health-related behaviors in youth during the COVID-19 pandemic.

Therefore, the primary objective of this study is to investigate the associations between PA, SB, and perceived stress among Korean adolescents. Specifically, we aimed to focus on changes in the perception of family ES during the COVID-19 outbreak in 2020, a period characterized by widespread implementation of social distancing measures and restrictions.

## 2. Materials and methods

### Study design

The KYRBS is a cross-sectional survey that was initiated in 2005 by the Korean Ministry of Education, the Ministry of Health and Welfare, and the Korean Centers for Disease Control and Prevention (KCDCP). The survey is designed to evaluate the risk of health-related behaviors in 15 areas (e.g., ES, PA, and mental health) among youths in South Korea. The validity and reliability of KYRBS’s questionnaire have been demonstrated in previous studies [30]. In this study, we assessed the variables of PA, SB, stress, and perception of changes in ES were assessed in adolescents aged 12–18 years who agreed to participate in the 16^th^ year (2020) of the KYRBS. Since this survey is an online anonymous survey that does not collect any personal information of the participants, it was not necessary to obtain ethical approval from an institutional review board (IRB). The data of KYRBS is the government-approved statistical survey (No. 117058) based on Article 18 of the Statistical Act of the Republic of Korea.

### Study participants

A total of 57,925 Korean adolescents from 800 schools (400 from middle schools and 400 from high schools) responded to the 2020 KYRBS survey. After excluding adolescents who did not respond and missing data, the final sample consisted of 54,948 participants. The current study divided the participants into two groups based on the response to the following question: “Do you think your family’s economic situation is deteriorating due to COVID-19 compared to before the pandemic?”. There were 4 possible answers (“very much”, “sort of”, “sort of not”, and “not at all”). Those who responded that their family’s ES had declined due to COVID-19 (i.e., very much) were categorized into the Declined ES (n = 3072), and those who responded that their family’s ES did not change (i.e., not at all) were categorized into the Non-changed ES (n = 3072). To ensure that the two groups were comparable, we excluded the ambiguous answers (i.e., sort of and sort of not), and the Non-changed ES group was matched in age, gender, and body mass index (BMI) to the Declined ES group. Therefore, a total of 6144 participants were included in this study.

### Measures

#### Physical activity

The daily PA was assessed using the following questions from the KYRBS. The moderate-intensity PA (MPA) was asked with the following question: “How many days do you usually do a total of 60 minutes or more of moderate-intensity PA (any type) for a week?”. An 8-point scale (i.e., 1 = Never, 2 = 1 day, 3 = 2 days, 4 = 3 days, 5 = 4 days, 6 = 5 days, 7 = 6 days, and 8 = every day) was added to the former question. Then, MPA minutes per week (mins/wk) were calculated by the following formula: the number of participation days x 60 minutes (i.e., minimum time spent in MPA). The vigorous-intensity PA (VPA) was asked with the following question: “How many days do you usually do 20 minutes or more of VPA (i.e., jogging, playing soccer, basketball, taekwondo, mountain climbing, riding a bicycle or swimming at high speed, and carrying heavy objects) for a week?”. Then, VPA minutes per week (mins/wk) were calculated by the following formula: the number of participation days x 20 minutes (i.e., minimum time spent in VPA). Lastly, participants’ MVPA levels (mins/wk) were calculated by combining MPA (mins/wk) and VPA (mins/wk). The participants were grouped into three categories of MVPA based on the current WHO PA guidelines of 60 minutes or more daily (i.e., 420 or more minutes per week) [15]: 1) ≥ 420 mins/wk, 2) < 420 mins/wk, and 3) Never (reference).

Muscular strength activities were asked with the following question: “How many days do you usually do muscular strength activities (i.e., push-ups, sit-ups, lifting weights, dumbbells, chin-up bar, and parallel bar) for a week?”. Further, a 6-point scale (i.e., 1 = Never, 2 = 1 day, 3 = 2 days, 4 = 3 days, 5 = 4 days, and 6 = More than 5 days) was added to the former questions. The participants were grouped into three categories based on the current WHO PA guidelines (i.e., at least 3 days per week) [15]: 1) ≥ 3 days/wk, 2) < 3 days/wk, and 3) Never (reference).

#### Sedentary behavior

The recreational SB variable was assessed by asking the following question: “How many hours do you usually sit in a day for non-learning purposes (i.e., sitting with friends, moving by car, bus, or train, reading a book, writing, playing a card game, watching TV, playing a video/computer game, and using the Internet)?”. The recreational SB was calculated as the amount of sitting time (minutes per week). Since there were no specific guidelines for SB from WHO, the participants were grouped into three categories of SB using Canadian 24-hour movement guidelines for children and youth (< 2 hrs/day), as recommended recreational sitting time cut-points [31]. Lastly, we converted the unit of cut-points from hours per day to minutes per week (i.e., < 840 mins/wk): 1) ≥ 840 mins/wk, 2) < 840 mins/wk, and 3) Never (reference).

#### Stress

The perception of stress was assessed through the following question: “How much stress do you usually feel in your daily life?”. A 5-point Likert Scale (i.e., 1 = Very Severe, 2 = Severe, 3 = Moderate, 4 = Mild, and 5 = Very Mild) was used for the perceived stress question.

#### Data analysis

All data obtained in this study were summarized by SPSS 26.0 version (SPSS Inc., Chicago, IL, USA). The participant’s personal information (i.e., age, education, actual family ES, and academic achievement) was examined by descriptive statistics, and anthropometrics information (i.e., height, weight, and BMI) was analyzed by an independent *t*-test to investigate the differences between the Declined ES group and the Non-changed ES group. Multinominal logistic regression was used to predict the impact of perception of family ES on the PA, SB, and stress variables, comparing the Declined ES group and the Non-changed ES group by adjusting the family’s socioeconomic status and participants’ academic achievement, which are measured categorically as responded by high, middle-high, middle, middle-low, and low. The results were presented as odds ratios (OR) with 95% confidence intervals (95% CI). Additionally, we conducted a comparative analysis by visually presenting the proportion of participants engaging in each PA, SB category, and stress levels for comparing the Declined ES group and the Non-changed ES group. All statistical significances were set by *p* < 0.05.

## 3. Results

Table 1 summarizes the participants’ demographic information. The study participants comprised 54.9% of boys and 45.1% of girls in middle or high school. The participants’ anthropometric information was presented as mean and standard deviation (SD). The mean height, weight, and BMI for boys were 171.24 ± 7.84 cm, 65.80 ± 13.78 kg, and 22.33 ± 3.91 kg·m^-2^, respectively, and the mean height, weight, and BMI for girls were 161.10 ± 5.38 cm, 53.86 ± 9.42 kg, and 20.72 ± 3.22 kg·m^-2^, respectively. The independent t-test results showed significant differences in participants’ age, height, weight, and BMI between the two groups (*p* < 0.05). The Declined ES group had a higher percentage of individuals with academic achievement in the low to middle level (67.5%), whereas the Non-changed ES group had more individuals in the middle to high level (77.1%). Additionally, the Declined ES group had a larger population of individuals in the low to middle classes of actual family ES (73.9%), while the Non-changed ES group had a higher representation in the middle to high classes (95.9%). A detailed description is presented in Table 1.

**Table 1:** Participants’ characteristics and anthropometrics information between the Declined ES group and Non-changed ES group.

Variable			Declined ES Group		Non-changed ES Group	
			(n = 3,072)		(n = 3,072)	
			No. (%)	Mean ± SD	No. (%)	Mean ± SD
Age (year)	Boys		1,760 (57.3)	15.15 ± 1.81	1,612 (52.5)	14.89 ± 1.78
	Girls		1,312 (42.7)	15.27 ± 1.78	1,460 (47.5)	14.85 ± 1.72
Anthropometrics	Boys	Height (cm)		171.31 ± 8.01		171.16 ± 7.66
		Weight (kg)		66.30 ± 14.60		65.29 ± 12.95
		BMI (kg·m^-2^)		22.46 ± 4.07		22.20 ± 3.74
	Girls	Height (cm)		160.99 ± 5.55		161.21 ± 5.20
		Weight (kg)		55.12 ± 10.11		52.65 ± 8.72
		BMI (kg·m^-2^)		21.22 ± 3.43		20.22 ± 3.00
Education	Middle School		1,545 (50.3)		1,845 (60.0)	
	High School		1,527 (49.7)		1,227 (40.0)	
Academic Achievement	High		383 (12.5)		665 (21.6)	
	Middle-High		615 (20.0)		897 (29.2)	
	Middle		774 (25.2)		809 (26.3)	
	Middle-Low		769 (25.0)		483 (15.7)	
	Low		531 (17.3)		218 (7.1)	
Family SES	High		331 (10.8)		829 (27.0)	
	Middle-High		471 (15.3)		1,183 (38.5)	
	Middle		1,109 (36.1)		933 (30.4)	
	Middle-Low		811 (26.4)		100 (3.3)	
	Low		350 (11.4)		27 (0.9)	

ES: Economic Status, SD: Standard Deviation, BMI: Body Composition Index; SES: Socioconomic Status; There are significant differences in age, height, weight, and BMI between the Declined ES group and the Non-changed ES group (*p* < .05).

Table 2 presents the findings of multinomial logistic regression analysis investigating the impact of perception of ES on PA, SB, and stress levels while adjusting for the family’s socioeconomic status and participants’ academic achievement. The results reveal notable differences between the Declined ES group and the Non-changed ES group. Regarding PA, adolescents who perceived a deteriorated family ES due to COVID-19 (i.e., Declined ES group) were 1.16 times more likely to engage in MVPA for less than 420 mins/wk compared to the Non-changed ES group (OR = 1.16; CI = 1.01–1.34; *p* = 0.039), but there was no significant difference in meeting recommended MVPA (i.e., ≥ 420 mins/wk) between the two groups (*p* > 0.05). Similarly, the odds of participation in muscular strength activities for 3 days or more (i.e., meeting recommendation) and for less than 3 days were 1.7 times (OR = 1.70; CI = 1.45–1.98; *p* < 0.001) and 1.36 times (OR = 1.36; CI = 1.18–1.58; *p* < 0.001) higher in the Declined ES group than the Non-changed ES group, respectively. Regarding SB, there were significant differences between the two groups. Adolescents in the Declined ES group were 37% less likely to spend time in sitting over 840 mins/wk (i.e., not meeting recommended recreational SB) (OR = 0.63; CI = 0.44–0.90; *p* < 0.001) and 43% less likely to spend time in sitting less than 840 mins/wk (OR = 0.57; CI = 0.41–0.79; *p* < 0.001) compared to the Non-changed ES group. Concerning stress levels, adolescents in the Declined ES group were 2.1 times more likely to experience a very severe level of stress (OR = 2.14; CI = 1.57–2.91; *p* < 0.001) and 42% less likely to experience a mild level of stress (OR = 0.58; CI = 0.44–0.78; *p* < 0.001) than their counterparts in the Non-changed ES group.

**Table 2:** Multinominal logistic regression was used to predict the impact of perception of family ES on the PA, SB, and stress variables comparing the Declined ES group and the Non-changed ES group.

Variable			Declined ES Group (n = 3,072)						
			*b*	S.E	*Wald*	*p*	*Exp(b)*	*Exp(b)* 95% Confidence Intervals	
								Lower	Upper
Physical Activity									
	MVPA								
		≥ 420 mins/wk	0.17	0.13	1.87	0.172	1.19	0.93	1.52
		< 420 mins/wk	0.15	0.07	4.26	0.039*	1.16	1.01	1.34
		Never	Ref						
	Muscular Strength								
		≥ 3 day	0.53	0.08	43.36	< .001***	1.70	1.45	1.98
		< 3 days	0.31	0.08	16.89	< .001***	1.36	1.18	1.58
		Never	Ref						
	Recreational SB								
		≥ 840 mins/wk	-0.46	0.18	6.62	0.010**	0.63	0.44	0.90
		< 840 mins/wk	-0.56	0.17	11.19	< .001***	0.57	0.41	0.79
		Never	Ref						
Stress									
		Very Severe	0.76	0.16	23.39	< .001***	2.14	1.57	2.91
		Severe	0.16	0.14	1.30	0.253	1.17	0.89	1.55
		Moderate	-0.13	0.14	0.84	0.358	0.88	0.68	1.15
		Mild	-0.54	0.15	13.45	< .001***	0.58	0.44	0.78
		Very Mild	Ref						

Reference group: Non-changed ES group; ***p < .001, **p < .01, *p < .05, OR: Odds Ratio, PA: Physical Activity, MVPA: Moderate-to-Vigorous Physical Activity (recommendation: 60 minutes or more daily (≥ 420 mins/wk)); Muscular strength: The number of days participating in muscular strength activities in the last 7 days (recommendation: at least 3 days per week); Each PA was categorized into 3 groups: Never, not meeting PA recommendation (< 420 mins/wk of MVPA; < 3 days/wk of muscular strength activities), meeting PA recommendation (≥ 420 mins/wk of MVPA; ≥ 3 days/wk of muscular strength activities); SB: A total minutes per week spent in sitting for non-learning purpose in the last 7 days; Recreational SB was categorized into 3 groups (recommendation: < 2hrs/day or < 840 mins/wk): ≥ 840 mins/wk, < 840 mins/wk, and Never.

The analysis was adjusted by family socioeconomic status and participants’ academic achievement (*p* < .001).

**Fig 1:**
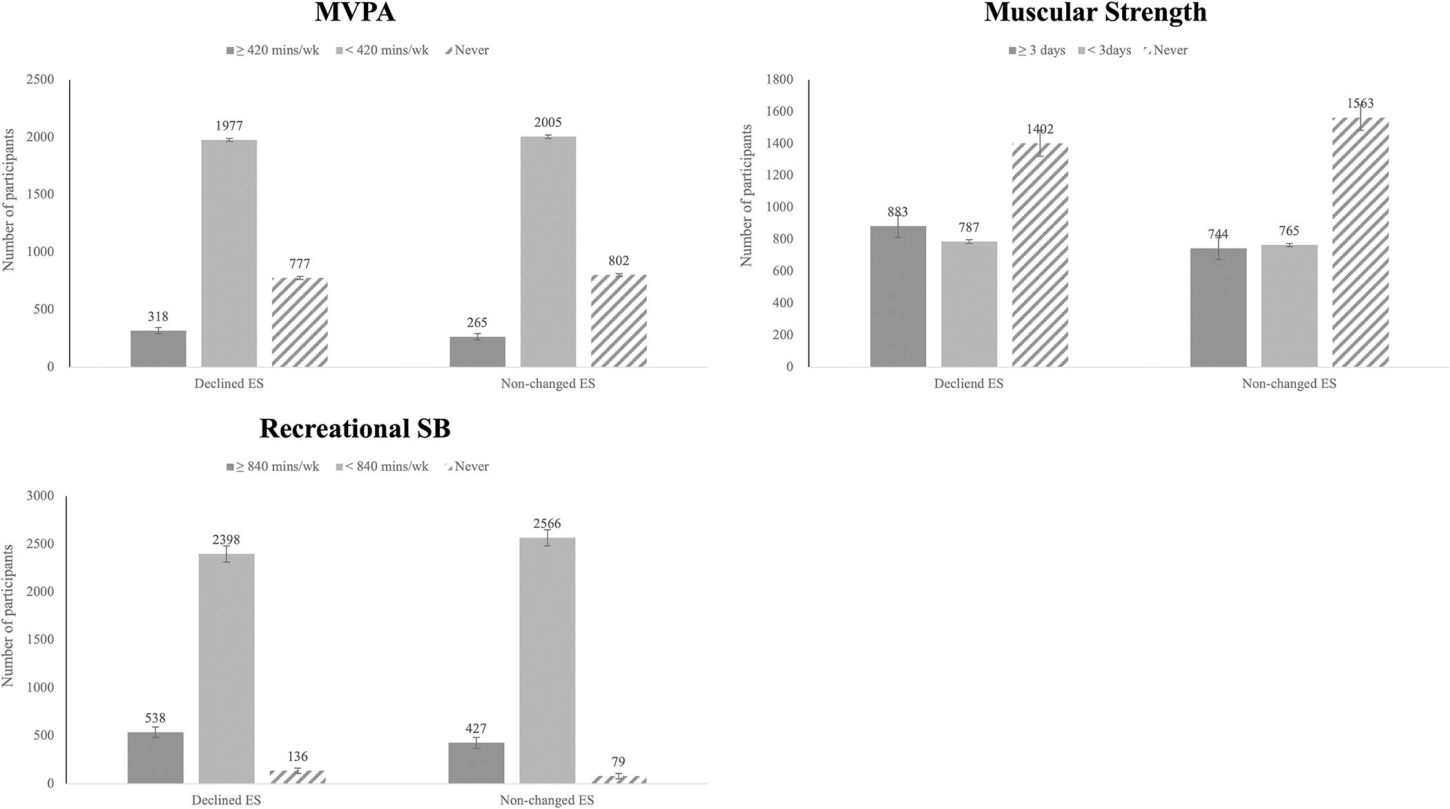
Comparison of physical activity and sedentary behavior between Declined ES and Non-changed ES groups. Each PA was categorized into 3 groups: Never, not meeting PA recommendation (< 420 mins/wk of MVPA; < 3 days/wk of muscular strength activities), meeting PA recommendation (≥ 420 mins/wk of MVPA; ≥ 3 days/wk of muscular strength activities); SB: A total minutes per week spent in sitting for non-learning purpose in the last 7 days; Recreational SB was categorized into 3 groups (recommendation: < 2hrs/day or < 840 mins/wk): ≥ 840 mins/wk, < 840 mins/wk, and Never.

Fig 1 presents the comparative analysis of the proportion of participants engaging in each PA, SB category, and stress levels for comparing the Declined ES group and the Non-changed ES group. In the Declined ES group (n = 3072), 318 individuals (10.4%) participated in MVPA more than 420 mins/wk (i.e., meeting recommendation [15]), 1977 individuals (64.4%) participated in MVPA less than 420 mins/wk and 777 individuals (25.3%) never participated in MVPA. In contrast, the Non-changed ES group (n = 3072) had 265 participants (8.6%) engaging in MVPA 420 minutes or more per week (i.e., meeting PA recommendation [15]), 2005 participants (65.3%) participated in MVPA less than 420 minutes per week, and 802 participants (26.1%) never participated in MVPA. For muscular strength activities, the Declined ES group had 883 individuals (28.7%) participating for 3 days or more per week (i.e., meeting recommendation [15]), 787 individuals (25.6%) for less than 3 days per week, and 1402 individuals (45.6%) never participated in muscular strength activities. The Non-changed ES group, on the other hand, had 744 participants (24.2%) for 3 days or more per week (i.e., meeting recommendation [15]), 765 participants (24.9%) for less than 3 days per week, and 1563 participants (50.9%) never participated in muscular strength activities. For recreational SB, the Declined ES group had 538 individuals (17.5%) spending time sitting 840 minutes or more per week (i.e., not meeting recommended recreational SB [31]), 2398 (78.1%) individuals for less than 840 minutes per week, and 136 individuals (4.4%) never spent time in sitting for non-learning purpose. The Non-changed ES group, on the other hand, had 427 individuals (13.9%) spending time in sitting 840 minutes or more per week (i.e., not meeting recommended SB [31]), 2566 individuals (83.5%) for less than 840 mins per week, and 79 individuals (2.6%) never spent time in sitting for non-learning purpose. Overall, both groups exhibited similar patterns in the proportion of participants engaging in MVPA and recreational SB. However, a larger proportion of participants in the Declined ES group met the recommendation for muscular strength activities (i.e., at least 3 days per week) compared to the Non-changed ES group.

**Fig 2:**
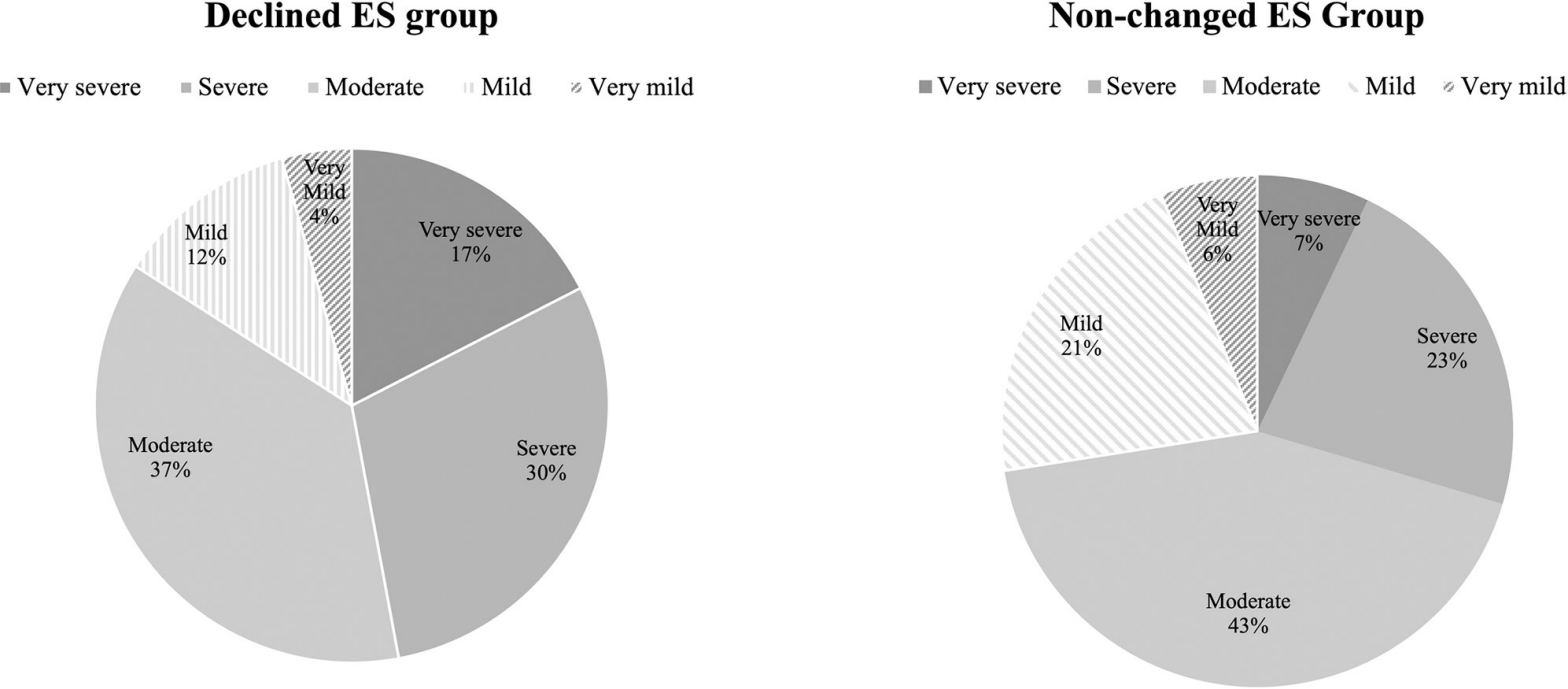
Comparison of stress level between Declined ES group and Non-changed ES group.

Fig 2 illustrates a graphical depiction of the daily stress levels comparison between the Declined ES group and the Non-changed ES group. In the Declined ES group, the highest proportion of participants reported experiencing a moderate level of stress, accounting for 37% of the sample. This was followed by 30% of participants reporting a severe level of stress and 17% reporting a very severe level of stress. In contrast, in the Non-changed ES group, the majority of participants (43%) reported experiencing a moderate level of stress. Subsequently, 23% of participants reported a severe level of stress, and 21 reported a mild level of stress.

## 4. Discussion

The outbreak of the COVID-19 pandemic brought about unprecedented challenges across various aspects of society, including the socioeconomic landscape of families. Our study delved into the intriguing interplay between adolescents’ perception of family ES changes due to the pandemic and its effects on adolescent PA, SB, and stress levels. While our initial hypothesis anticipated a certain trajectory, the unexpected findings shed light on the complex ways in which adolescents respond to adversity.

Contrary to our expectations, adolescents in the Declined ES group were more likely to engage in MVPA and muscular strength activities, less likely to spend time in sitting, and more likely to experience a very severe stress level compared to the Non-changed ES group. One of the most intriguing outcomes of our study was the increased engagement in MVPA and muscular strength activities among adolescents in the Declined ES group, which is inconsistent with previous studies that individuals with high ES are more physically active than those who are with low ES [32, 33]. Although there was no significant difference in terms of meeting the recommended MVPA duration of 60 minutes or more daily (i.e., ≥ 420 mins/wk) between the two groups, adolescents in the Declined ES group were 1.2 times more likely to engage in MVPA for less than 420 mins/wk (OR = 1.16, *p* = 0.039), 1.7 times more likely to participate in muscular strength activities for more than 3 days/wk (i.e., meeting recommendation; OR = 1.70, *p* < 0.001), and 1.4 times more likely to participate in muscular strength activities less than 3 days/wk (OR = 1.36, *p* < 0.001) compared to the Non-changed ES group. These results suggest a plausible interpretation that adolescents in the Declined ES group seemed to channel their energies toward physical pursuits in the face of economic uncertainty and upheaval. This shift in activity patterns could be attributed to various factors, including a desire to cope with stress or a need for a productive outlet, using increased leisure time due to changes in routine during the pandemic [34–37].

Simultaneously, alongside their increased engagement in PA, adolescents in the Declined ES group exhibited a decrease in recreational sedentary time (≥ 840 mins/wk: OR = 0.63, *p* < 0.001; < 840 mins/wk: OR = 0.57, *p* < 0.001). These findings also presented a departure from prior research, which had previously established an inverse correlation between SB and ES in adolescents [38]. However, this reduction of recreational SB could potentially reflect the altered nature of daily life during pandemic-related lockdowns and the widespread adoption of remote learning, which led to increased leisure time activities [36]. The shift away from excessive SB might further underscore the adaptability of adolescents in the face of extraordinary circumstances during the pandemic (e.g., the perception of family ES changing to deteriorate), but further research is needed to understand the association between the perception of ES changes and behavioral changes among adolescents.

Concerning stress levels, adolescents in the Declined ES group manifested a 2.14-fold higher likelihood of encountering very severe levels of stress (OR = 2.14, *p* < 0.001) than their counterparts in the Non-changed ES group. This could be attributed to the myriad of uncertainties and disruptions brought on by the pandemic, including concerns about family financial stability and educational continuity. In addition, these escalated stress levels among adolescents can be attributed to a multitude of factors, encompassing economic deterioration resulting in heightened financial burdens, adverse shifts in parental mental health, disruptions in marital interaction, and potential declines in parenting quality [39]. It suggests that the imposition of economic constraints during the COVID-19 pandemic likely accentuated these stressors, disproportionately affecting the Declined ES group in our study, who perceived a deterioration in their family ES. However, it is noteworthy that the adolescents in the Non-changed ES group also exhibited a significant increase in a mild level of stress compared to adolescents in the Declined ES group. This indicates that the pandemic’s impact on stress was not solely driven by perception of ES changes in adolescents, but likely influenced by broader contextual factors. Consequently, further investigations are necessary to explore additional pertinent factors that could influence stress levels among adolescents.

It is widely acknowledged that socioeconomic status is closely linked to health outcomes [40], and numerous studies have explored the association between ES and health-related behaviors, particularly in relation to PA or SB [41, 42]. In our study, we observed that the Declined ES group had a larger population of individuals in the low to middle classes of family socioeconomic status (73.9%), while the Non-changed ES group had a higher representation in the middle to high classes (95.9%). Based on this observation, we propose that adolescents from low socioeconomic status families were more likely to perceive deteriorated changes in their family ES compared to those from high socioeconomic status families during the COVID-19 pandemic. This perception of the economic downturn may have impacted behavioral changes and increased stress levels among adolescents in the Declined ES group. Although we accounted for family socioeconomic status as a covariate for adjustment using categorical variables (i.e., high, middle-high, middle, middle-low, and low), it is essential for future research to incorporate more precise measures of parents’ actual income levels. In addition, to ensure a comprehensive comprehension of the association observed, it is imperative to note that our analysis incorporated academic achievement as a covariate, which is one of the potential confounders that can affect the associations between the perception of changes in ES and behavior changes [43–45]. Nonetheless, future investigations should account for other pertinent factors related to ES, health-related behaviors (i.e., PA and SB), and stress levels in adolescents.

Concluding the discourse, it is noteworthy that adolescents, due to their sensitive and critical developmental stage, are particularly susceptible to the deleterious effects of chronic stress [46]. Notably, Kong et al. reported that stress could profoundly impact both the physical and mental health of individuals, contributing to conditions such as depression, suicide, sleep disturbances, cardiovascular disease, and compromised immune function [46]. Concurrently, extant research has demonstrated the beneficial role of PA and exercise in promoting mental health [47–49]. Although adolescents in the Declined ES group in our study showed increased PA and reduced SB while increased severe stress levels, it is essential to provide mental health care for adolescents who are susceptible to perceived deteriorated ES during the pandemic as the increased stress level is a negative indicator of adolescents’ mental health. Furthermore, previous studies have focused only on the association between deteriorated economic situation and health status among adolescents [50–52]. However, considering that adolescents in the Non-changed ES group in our study exhibited less engagement in PA, high involvement in SB, and increased a mild level of stress compared to adolescents compared to the Declined ES group, it is also necessary to focus on the negative impact on health behavior of adolescents even if there is no deterioration on family ES during the pandemics.

### Strength and limitations

The present study demonstrates several noteworthy strengths. Firstly, it represents the inaugural investigation to explore the association between PA, SB, and stress, particularly among age-matched adolescents, by categorizing the cohorts based on their perception of changes in family ES during the COVID-19 pandemic. Furthermore, our research yields novel findings by analyzing significant disparities in PA, SB, and stress among adolescents, taking into account their perception of family ES during the COVID-19 period. Nonetheless, the study is not without its limitations, which necessitate acknowledgment. First, the variable representing their perception of changes in family ES due to COVID-19 was subjectively answered by adolescents, which may lead to recall bias. Additionally, the data on PA and SB were derived from self-reported responses, potentially leading to either underestimation or overestimation by individuals [53]. Furthermore, the study did not incorporate the actual income level of parents due to the lack of accurate specifications for categorizing their income levels in the survey. To improve the validity of study results, future research should utilize reliable data on objectively measured PA and the actual family income levels as a socioeconomic status measure.

## 5. Conclusion

In conclusion, the study has identified notable variations in levels of PA, SB, and stress levels among Korean adolescents, contingent on their perception of family ES in the aftermath of the COVID-19 outbreak in 2020. These findings offer valuable insights into the potential significance of promoting physical and mental health care among adolescents to bolster their physical and mental well-being during future pandemics, regardless of the perception of changes in ES. Based on the findings, we propose the implementation of targeted policies for improving mental health aimed at adolescents from low ES families or those susceptible to experiencing a decline in family ES following the future disease pandemic. Moreover, to enhance comprehension, it is crucial to gather further evidence from diverse countries worldwide to investigate the association between PA, SB, mental health, ES, and perception of ES among adolescents during disease pandemics.
